# Effects of the Neurogranin Variant rs12807809 on Thalamocortical Morphology in Schizophrenia

**DOI:** 10.1371/journal.pone.0085603

**Published:** 2013-12-30

**Authors:** Jamie Yu Jin Thong, Anqi Qiu, Min Yi Sum, Carissa Nadia Kuswanto, Ta Ahn Tuan, Gary Donohoe, Yih Yian Sitoh, Kang Sim

**Affiliations:** 1 Department of Bioengineering, National University of Singapore, Singapore; 2 Clinical Imaging Research Center, National University of Singapore, Singapore; 3 Singapore Institute for Clinical Sciences, the Agency for Science, Technology and Research, Singapore; 4 Research Division, Institute of Mental Health, Singapore; 5 Department of Psychiatry, School of Medicine and Trinity College Institute of Neuroscience, Trinity College Dublin, Dublin, Republic of Ireland; 6 Department of Neuroradiology, National Neuroscience Institute, Singapore; 7 Department of General Psychiatry, Institute of Mental Health, Singapore; University of Illinois at Chicago, United States of America

## Abstract

Although the genome wide supported psychosis susceptibility neurogranin (NRGN) gene is expressed in human brains, it is unclear how it impacts brain morphology in schizophrenia. We investigated the influence of NRGN rs12807809 on cortical thickness, subcortical volumes and shapes in patients with schizophrenia. One hundred and fifty six subjects (91 patients with schizophrenia and 65 healthy controls) underwent structural MRI scans and their blood samples were genotyped. A brain mapping algorithm, large deformation diffeomorphic metric mapping, was used to perform group analysis of subcortical shapes and cortical thickness. Patients with risk TT genotype were associated with widespread cortical thinning involving frontal, parietal and temporal cortices compared with controls with TT genotype. No volumetric difference in subcortical structures (hippocampus, thalamus, amygdala, basal ganglia) was observed between risk TT genotype in patients and controls. However, patients with risk TT genotype were associated with thalamic shape abnormalities involving regions related to pulvinar and medial dorsal nuclei. Our results revealed the influence of the NRGN gene on thalamocortical morphology in schizophrenia involving widespread cortical thinning and thalamic shape abnormalities. These findings help to clarify underlying NRGN mediated pathophysiological mechanisms involving cortical-subcortical brain networks in schizophrenia.

## Introduction

Schizophrenia is a heterogeneous psychiatric disorder with a complex etiology. There is a strong genetic component involved in the pathogenesis of schizophrenia. In recent years multiple genetic markers have been identified as conferring increased risk for schizophrenia from genome wide association studies [1,2]. One of these markers is the rs12807809 (T/C) single nucleotide polymorphism (SNP) in the neurogranin (NRGN) gene [2]. The NRGN gene is localized on chromosome 11q24.2, contains 4 exons and 3 introns and the 78 amino acid protein is encoded by part of exon 1 and 2 [3]. The NRGN protein is a postsynaptic protein that is expressed in the human brain and involved in the regulation of calmodulin availability in neurons [4,5]. NRGN has been implicated with important roles in synaptic signaling, plasticity, neural development, learning and memory [6,7]. 

Extant sparse structural and functional neuroimaging studies which sought to correlate the effect of the NRGN gene on brain structure or function have found changes of brain activations involving the frontal cortex, medial temporal lobe and cingulate cortices in healthy controls [8,9,10]. Pohlack and colleagues [9] reported reductions in hippocampal function in individuals who were risk T-allele homozygote during the acquisition phase of a contextual fear paradigm whilst Krug et al. (2011) [8] found that the risk T-allele homozygote showed differential activation patterns of the anterior and posterior cingulate cortices during an episodic memory task compared with C-allele carriers. In a recent study combining structural and functional magnetic resonance neuroimaging [10], it was noted that there was no NRGN rs12807809 effect on brain structures or functions. However, C-allele carriers showed a load independent decrease of brain activity in the left superior frontal gyrus within a working memory task but not individuals who were risk T-allele homozygotes, suggesting that the risk TT genotype may mediate reduction in processing efficiency within the frontal lobe networks. In the only imaging-genetic study involving patients with schizophrenia, Ohi and colleagues [11] found that patients with schizophrenia who are risk T-allele carriers had smaller left anterior cingulate grey matter volumes.

To the best of our knowledge, there is no study examining the impact of putative genome wide supported psychosis susceptibility NRGN gene on neural substrates such as cortical thickness and other subcortical brain structures such as amygdala, thalamus, basal ganglia in patients suffering from schizophrenia. First, this is relevant in the context of recent meta-analytic data [12] which highlighted that genetic risk for schizophrenia is still better indexed by brain structure and function rather than by other measures such as cognitive measures. Second, it is thought that as brain grey matter volume is a composite of surface area measurements and cortical thickness with different genetic influences, the latter parameters such as cortical thickness are hence preferred over grey matter volumes for determination of genetic effects on brain structures [13]. Third, elucidation of changes in cortical thickness and subcortical volumetric and shape changes together would allow insights into any disruptions of cortical-subcortical circuitry and genes mediating these brain structural changes. Thus in this study, we aimed to study the influence of NRGN rs1280709 on cortical thickness and volumes and shapes of subcortical structures in patients with schizophrenia and in healthy subjects. Based on previous data of frontal-limbic and fronto-thalamic circuitry disturbances in schizophrenia [14,15] and sparse data relevant to NRGN effect in psychosis, we hypothesized that patients with schizophrenia with risk T homozygote genotype were associated with cortical thinning involving frontal, temporal regions as well as subcortical structural abnormalities implicating the thalamus and hippocampus. 

##  Methods

### Subjects

The study was approved by the National Healthcare Group Institutional Review Board as well as the Institutional Review Board of the National Neuroscience Institute, Singapore. All subjects gave their written informed consent following a complete description of the study.

A total of one hundred and fifty six subjects of Chinese ethnicity were recruited for the study. Subjects with schizophrenia (SCZ) were recruited from the Institute of Mental Health, Singapore, while healthy comparison subjects (CON) were recruited from the community via advertisements. 

Ninety one patients with a DSM-IV diagnosis of schizophrenia participated this study. Confirmation of the diagnosis was made for all patients by a psychiatrist using information obtained from the patient’s clinical history, existing medical records, interviews with significant others as well as the administration of the Structured Clinical Interview for DSM-IV disorders-Patient Version (SCID-I/P). The patients were maintained on a stable dose of antipsychotic medications (60 on second generation antipsychotics, 28 on first generation antipsychotics and 3 on a combination of first and second generation antipsychotics; mean daily chlorpromazine equivalents of 242 mg (range 30 to 600 mg) for at least two week and did not have their medications withdrawn for the purpose of the study. None of the subjects had any history of any significant neurological illness such as epilepsy, head trauma, or cerebrovascular accidents. No subject met DSM-IV criteria for alcohol abuse or other substance abuse within the preceding three months of their recruitment into the study. 

Sixty five healthy comparison subjects were recruited and screened using the SCID Non-Patient version (SCID-I/NP) to ensure that they did not have any Axis I psychiatric disorder. None of them had any history of a major neurological illness, medical illnesses, substance abuse or psychotropic medication use. 

Both patients and controls were further split into groups based on their genotype (TT vs TC+CC). In total, 51 patients and 34 controls were T-allele homozygotes while 40 patients and 31 controls were C-allele carriers.

### Clinical Measures

The Positive and Negative Syndrome Scale (PANSS) was used to assess psychopathology and symptom severity, while the Global Assessment of Functioning (GAF) Scale was used to assess social, occupational and psychological functioning. Both scales were administered by a psychiatrist to all the participants. Table **1** summarizes the demographic characteristics of each of the 4 groups and the clinical scores for the SCZ groups. 

**Table 1:** Demographics and clinical features of the sample.

	CON T-allele Homozygotes (n=34)	CON C-allele carriers (n=31)	SCZ T-allele homozygotes (n=51)	SCZ C-allele carriers (n=40)	p-value
Age (years)	36.6±11.2	36.6±10.3	38.3±9.72	38.6±9.72	0.764
Gender (Female/Male)	22/12	20/11	40/11	28/12	0.452
Handedness (% right)	94%	90%	94%	83%	0.238
Education (years)	13.7±1.91	14.1±2.14	11.8±1.86	11.1±2.72	**<0.001**
Mean Illness Duration (years)	-	-	7.96±7.90	6.70±8.05	0.455
Antipsychotic dose (mg CPZ equivalents)	-	-	219.1±198.3	220.6±175.9	0.967
PANSS total scores	-	-	38.0±7.45	40.6±11.2	0.195
GAF score	-	-	52.4±17.0	49.8±19.0	0.486

Note: CON – control; SCZ – schizophrenia; CPZ – Chlorpromazine; PANSS – Positive and Negative Syndrome Scale; GAF – Global Assessment of Functioning. Significant p-values are denoted in bold font.

### Image acquisition and Analysis

High-resolution T1-weighted Magnetization Prepared Rapid Gradient Recalled Echo (MPRAGE, TR=7.2s; TE=3.3ms; flip angle=8°, field of view=230 mm × 230 mm; acquisition matrix=256 × 256) images were acquired at the National Neuroscience Institute, Singapore, on a 3-Tesla whole body scanner (Philips Achieva, Philips Medical System, Eindhoven, The Netherlands) with a SENSE head coil. Stability of a high signal to noise ratio was assured through a regular automated quality control procedure. 

The gray matter, white matter, cerebral spinal fluid (CSF), lateral ventricles, and subcortical structures (amygdala, hippocampus, caudate, putamen, globus pallidus, thalamus) were automatically segmented from the intensity-inhomogeneity corrected T1-weighted MR images [16]. The subcortical and lateral ventricular volumes were calculated from the segmented image. For subcortical shape analysis, the subcortical and lateral ventricular structures were generated using the prior shape information of an atlas that were created from 41 manually labeled individual structures via a LDDMM atlas generation procedure [17]. Shape variations of individual subjects relative to the atlas were characterized by the Jacobian determinant of the deformation in the logarithmic scale, where the deformation transformed the atlas shape to be similar to subjects. This measure, termed as the “deformation map”, represents the ratio of each subject’s structural volume to the atlas volume in the logarithmic scale: i.e. positive values correspond to expansion, while negative values correspond to compression of the subject’s structure relative to the atlas at each anatomical location.

For cortical thickness, an inner surface was constructed at the boundary between WM and GM and then propagated to the outer surface at the boundary between GM and CSF. The cortical thickness was measured as the distance between the corresponding points on the inner and outer surfaces [18]. A cortical surface mapping algorithm, large deformation diffeomorphic metric mapping (LDDMM), was then applied to align individual cortical surfaces to an atlas cortical surface for group analysis of cortical thickness [19].

### Genotyping Procedures

 Genotyped data (SNP rs12807809) were obtained through an ongoing genetic association study using the Illumina HumanHap 250K and 317K Beadchips (Illumina Inc., San Diego, USA) at the Genome Institute of Singapore, Agency for Science, Technology and Research. The DNA sample was isothermally amplified to be subsequently fragmented by a controlled enzymatic process that does not require gel electrophoresis. The DNA was consequently alcohol precipitated, resuspended and hybridized. Allelic specificity was conferred by enzymatic single-based extension reaction followed by fluorescence staining. The intensities of the beads’ fluorescence were picked up by the Illumina BeadArray Reader and analyzed using Illumina BeadStudio software. Single nucleotide polymorphisms (SNPs) that lie within the NRGN gene locus were identified from the Database of Single Nucleotide Polymorphisms (dbSNP, available from: http://www.ncbi.nlm.nih.gov/SNP/). These were then matched with the marker list from Illumina to obtain the SNP of interest, rs12807809 (ss20856537) for analysis. For quality control, the samples were only included for further analysis if the genotyping rate was >98%, call rate >90%, a minor allele frequency (MAF) > 5% and the samples were in Hardy-Weinberg equilibrium (HWE p > 0.05). Statistical analyses were performed using the Haploview v4.2 (Barrett et al., 2005) and PASW18.

### Statistical Analysis

Demographic and clinical characteristics among the four groups of CON T-allele homozygotes, CON C-allele carriers, SCZ T-allele homozygotes, SCZ C-allele carriers were compared using chi-square tests for categorical variables and ANOVA for continuous variables. Post-hoc analysis for continuous variables was conducted with Bonferroni’s corrections for multiple comparisons. ANOVA was also used to examine group differences in total brain volume (TBV) and subcortical volumes with Bonferroni’s test for multiple comparisons, with a p value < 0.0026 (0.05/19 structural volumetric measures) considered as the threshold for significance.

We first examined the interaction effect between diagnosis and genotype on subcortical shapes and cortical thickness. For this, the subcortical deformation maps and cortical thickness maps were smoothed using a 30 mm full-width-at-half-maximum Gaussian filter [20]. Linear regression with diagnosis and genetic type and their interaction as the main factors was examined at each vertex. Results at each surface vertex were thresholded at the level of significance (p<0.001) and then corrected for multiple comparisons at the cluster level of significance (p<0.05). Each cluster size must be greater than 358mm^2^, which was determined based on random field theory [21]. We additionally examined pairwise group difference in subcortical shapes and cortical thickness using linear regression with groups (C-allele control carriers, T-allele control homozygotes, C-allele schizophrenia carriers, and T-allele schizophrenia homozygotes) as the main factor. Further analysis was conducted when years of education was considered as covariate.

##  Results

### Sample Demographics and Clinical Features

The ratio of T-allele homozygotes to C-allele carriers is 54.5% to 45.5%, which is more balanced than the allele frequency distributions in Krug et al. (68.9% T-allele homozygotes), Pohlack et al. (67.9% T-allele homozygotes) or Rose et al. (72.1% T-allele homozygotes) [8,9,10]. 

No group differences were found in age (F_3,152_ = 0.41, *p* = 0.746), sex (χ^2^ = 2.63, *p* = 0.452), or handedness (χ^2^ = 4.22, *p* = 0.238) among CON T-allele homozygotes, SCZ T-allele homozygotes, CON C-allele carriers, and SCZ C-allele carriers. These variables were therefore not included as covariates in our subsequent analysis. Group differences in years of education were found, (F_3,152_ = 16.53, *p* < 0.001), with both patient groups having fewer years of education than the control groups. The mean difference in years between SCZ T-allele homozygotes and CON T-allele homozygotes and C-allele carriers was 1.94 ± 0.48 (p = 0.001) and 2.36 ± 0.50 (p < 0.001) respectively, while the mean difference between the SCZ C-allele carriers and the CON T-allele homozygotes and CON C-allele carriers were 2.58 ± 0.51 (p < 0.001) and 3.00 ± 0.52 (p < 0.001) respectively. There were no group differences found in the PANSS total score (F_1,89_ = 1.70, *p* = 0.195), GAF score (F_1,89_ = 0.49, *p* = 0.486), duration of illness (F_1,89_ = 0.56, *p* = 0.455), or antipsychotic dose (F_1,89_ = 0.002, *p* = 0.967) between SCZ T-allele homozygotes and SCZ C-allele carriers.

### Total Brain Volume, Gray and White Matter Volumes and Subcortical Volumes

 Our analysis did not reveal any interaction effects of diagnosis and genotype on subcortical volumes after correcting for multiple comparisons (Table **2**). For pairwise group comparisons, no group differences were found in any structures except for the lateral ventricles after correcting for multiple comparisons (Table **2**). There was a diagnosis effect on the left ventricle volume (F_3,152_ = 10.513, *p* = 0.001) and right ventricle volume (F_3,152_ = 12.758, *p* < 0.001), with larger ventricular volumes in the SCZ group. These results remain unchanged after controlling for years of education.

**Table 2:** The effects of diagnosis and genotype on brain volumes.

		**CON (65)**				**SCZ (91)**				**ANOVA**							
		**TT**		**C carriers**		**TT**		**C carriers**		**Diagnosis effect**			**Genotype effect**			**Interaction**	
**Structure Volumes**		**Mean ± Standard Deviations**								***F***	***P***		***F***	***P***		***F***	***P***
Total Brain (ml)		1107±118		1096±90		1099±86		1063±98		1.606	0.207		2.262	0.135		0.650	0.421
Left Gray Matter (ml)		226±26		218±20		218±19		210±19		5.721	0.018		4.764	0.031		0.008	0.929
Right Gray Matter (ml)		225±26		219±21		218±19		210±19		5.051	0.026		3.821	0.052		0.102	0.750
Left White Matter (ml)		235±28		236±23		237±21		229±26		0.343	0.559		0.661	0.417		1.078	0.301
Right White Matter (ml)		235±28		237±24		237±23		230±26		0.396	0.530		0.490	0.485		1.208	0.273
Left Amygdala (mm^3^)		1520±276		1547±298		1429±380		1391±347		5.078	0.026		0.009	0.925		0.352	0.554
Right Amygdala (mm^3^)		1796±266		1734±331		1647±390		1625±307		5.622	0.019		0.584	0.446		0.134	0.714
Left Caudate (mm^3^)		3064±865		3372±670		3396±629		3274±621		1.072	0.302		0.678	0.412		3.623	0.059
Right Caudate (mm^3^)		3046±732		3359±598		3306±682		3150±712		0.052	0.820		0.493	0.484		4.386	0.038
Left Hippocampus (mm^3^)		3806±454		3784±488		3671±634		3643±372		2.772	0.098		0.090	0.764		0.001	0.973
Right Hippocampus (mm^3^)		3943±468		3720±624		3767±694		3763±489		0.486	0.487		1.405	0.238		1.306	0.255
Left Pallidus (mm^3^)		1745±284		1777±208		1898±231		1858±250		8.728	0.004		0.010	0.922		0.810	0.370
Right Pallidus (mm^3^)		1675±253		1830±662		1787±375		1713±242		0.002	0.966		0.387	0.535		3.045	0.083
Left Putamen (mm^3^)		6162±1087		6376±747		6486±764		6332±719		1.067	0.303		0.047	0.828		1.846	0.176
Right Putamen (mm^3^)		6288±750		5791±1404		6233±1133		6217±662		1.234	0.268		2.367	0.126		2.074	0.152
Left Thalamus (mm^3^)		6691±863		7002±727		6756±638		6687±708		5.046	0.021		0.060	0.807		0.116	0.734
Right Thalamus (mm^3^)		6876±842		6903±736		6654±613		6526±701		6.621	0.011		0.186	0.667		0.447	0.505
Left Ventricle (mm^3^)		8272±4030		9125±2993		10881±3951		10980±5166		10.513	**0.001**		0.478	0.490		0.300	0.585
Right Ventricle (mm^3^)		7604±3550		7984±2388		9882±3086		9620±3924		12.758	**< 0.001**		0.011	0.915		0.343	0.559

Note: CON – control; SCZ – schizophrenia.

### Effect of Risk Allele on Cortical Thickness

**Figure 1:**
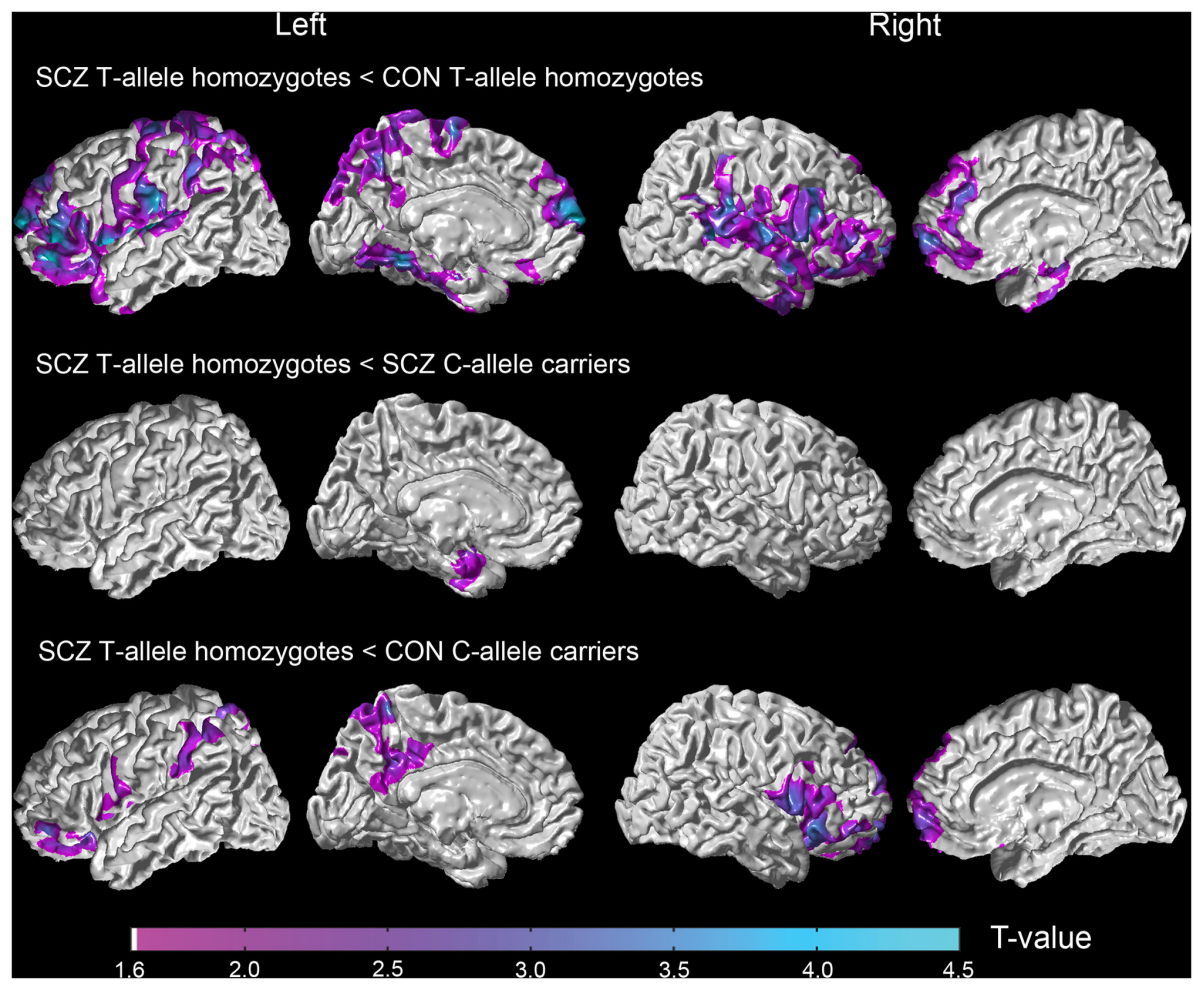
Statistical maps of cortical thickness differences between schizophrenia T-allele homozygotes and: control T-allele homozygotes (top panel); schizophrenia T-allele homozygotes and schizophrenia C-allele carriers (middle panel); schizophrenia T-allele homozygotes and control C-allele carriers (bottom panel). T-values are shown only in the regions with significant group differences after correction for multiple comparisons. Keys: SCZ – Schizophrenia; CON – Control.

No interaction effects of diagnosis and genotype on cortical thickness after correcting for multiple comparisons. However, Figure **1** and Figure **S1** in the File S1 show the results of pairwise group comparisons on cortical thickness. Differences in cortical thickness were most widespread in SCZ T-allele homozygotes when compared to CON T-allele homozygotes (Figure **1** **, top panel**). Specifically, SCZ T-allele homozygotes had thinner cortex across wide areas of the frontal, temporal and parietal lobes. Areas affected bilaterally included the dorsolateral prefrontal cortex, orbitofrontal gyrus, superior frontal gyrus, inferior frontal gyrus, temporopolar area, fusiform gyrus, entorhinal cortex, primary and auditory association cortices, the anterior and posterior regions of the rostral medial frontal cortex, and the supramarginal gyrus. In the left hemisphere, thinner cortex was found in the primary motor cortex, primary somatosensory cortex, somatosensory association cortices, and orbital medial frontal cortex. In the right hemisphere, the middle temporal gyrus and the entire superior temporal gyrus were thinner in the SCZ T-allele homozygotes compared with CON T-allele homozygotes.

 Compared to SCZ C-allele carriers (Figure **1** **, middle panel**), SCZ T-allele homozygotes had thinner cortex in the left entorhinal cortex. Compared to CON C-allele carriers (Figure **1** **, bottom panel**), SCZ T-allele homozygotes had thinner cortex bilaterally in the orbitofrontal cortex and inferior frontal gyrus, the isthmus, supramarginal gyrus, superior parietal gyrus and precuneus in the left hemisphere and the orbital medial frontal cortex, frontal pole and superior frontal gyrus in the right hemisphere. The other group comparisons are reported in the Summplement.

 We repeated the analysis for the above comparisons while controlling for years of education. Our results remain largely unchanged. 

### Effect of Risk Allele on Subcortical Shapes

**Figure 2:**
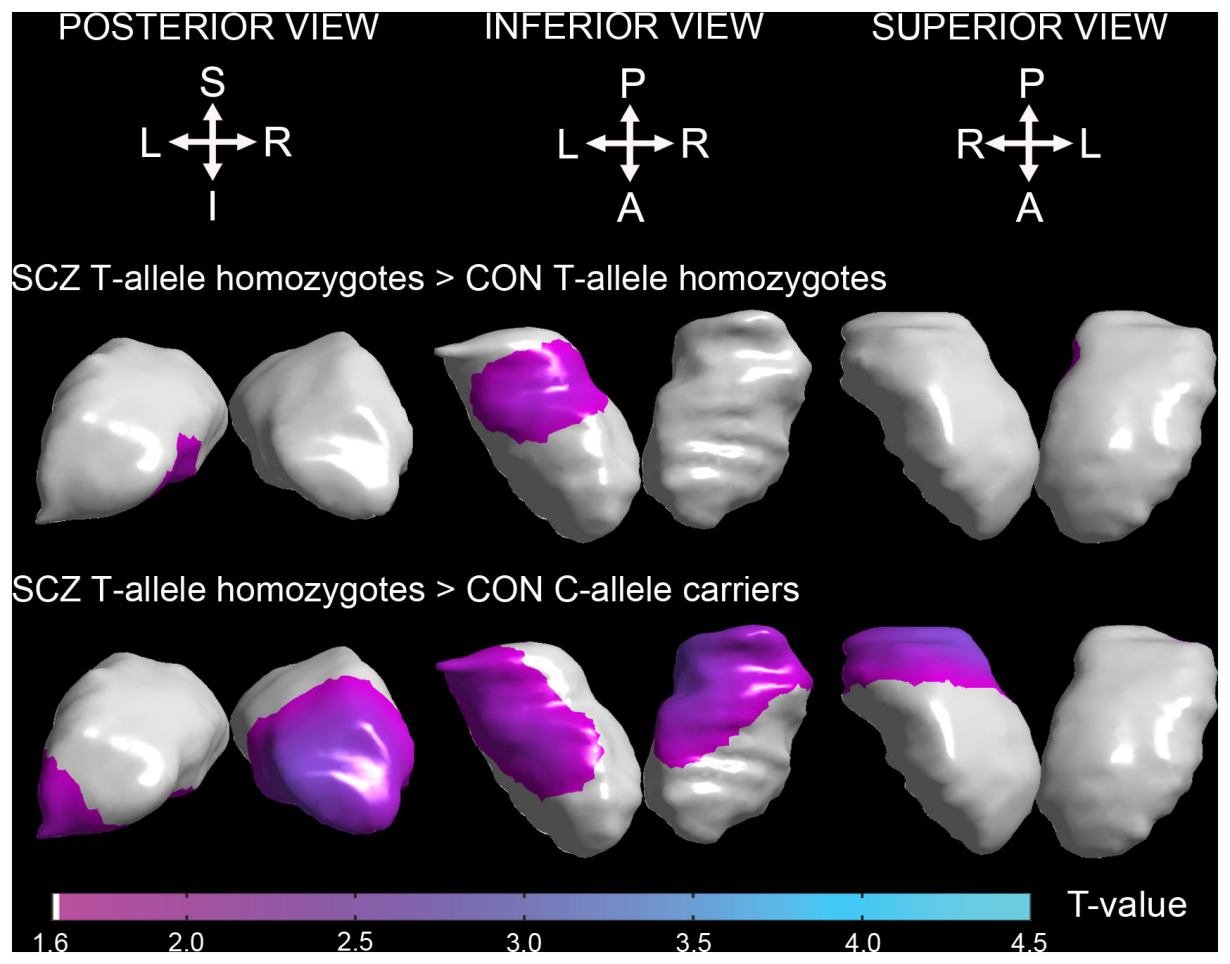
Statistical maps of thalamic shape differences between schizophrenia T-allele homozygotes and control T-allele homozygotes (top panel); schizophrenia T-allele homozygotes and control C-allele carriers (middle panel). T-values are shown only in the regions with significant group differences after the correction of multiple comparisons. Keys: S – superior; I – inferior; A – anterior; P – posterior; L – left; R – right.

No interaction effects of diagnosis and genotype on subcortical shapes after correcting for multiple comparisons. However, significant shape differences between the groups were found in the thalamus (**Figure 2 and Figure S2 **in File **S1**). When compared to CON T-allele homozygotes, inward-surface deformation was observed in the medial-inferior aspect of the left thalamus, mostly corresponding to the medial-inferior aspect of the left pulvinar nucleus in SCZ T-allele homozygotes (Figure **2** **, top panel**). Compared to CON C-allele carriers, SCZ T-allele homozygotes showed more widespread inward-surface deformation across the inferior and posterior aspects of the thalamus bilaterally and the medial aspect of the right thalamus (Figure **2** **, middle panel**). These regions mostly correspond to the pulvinar, ventral and mediodorsal nuclei of the thalamus. No group difference in the thalamic shapes was found between SCZ T-allele homozygotes and SCZ C-allele carriers. The other group comparisons are reported in the File S1.

We repeated the analysis for the above comparisons while controlling for years of education. Our results remain largely unchanged. 

## Discussion

Although interactive effects of diagnosis and genetic type on cortical thickness and subcortial shapes were not found, there were several significant findings in this study. First, schizophrenia T-allele homozygotes had widespread cortical thinning involving frontal, parietal and temporal cortices compared with control T-allele homozygotes. Second, no volumetric difference in subcortical structures (hippocampus, thalamus, amygdala, basal ganglia) was observed between risk T-allele homozygote genotype in patients with schizophrenia and controls. Third, risk T-allele homozygote genotype was associated with thalamic shape abnormalities involving regions related to pulvinar and medial dorsal nucleus in patients with schizophrenia compared to controls. These findings suggest that NRGN related brain structural abnormalities are found involving thalamocortical circuitry. 

Our findings of cortical thinning involving frontal, temporal and parietal cortices are consistent with cortical thickness changes which have been previously reported in schizophrenia at different phases of illness including first episode cases [22,23] as well as in those with chronic illness [24]. Crespo-Facorro et al. [22] reported significant total cortical thinning and especially involving frontal, temporal and parietal cortices in their study of 142 patients with first episode schizophrenia and compared with 83 healthy controls. This was consistent with other studies of similar patient groups and involving thinning of fronto-temporal-parietal cortical regions [25,26]. Widespread cortical thinning involving frontal, temporal and parietal regions were found in patients with chronic schizophrenia, highlighting that schizophrenia is associated with a potential neurodevelopmental deficits involving disruption of cortical maturation [27,28]. In addition, these changes in cortical thickness have been found to progress over time [29,30]. Cobia et al. [29] found in their 2 year follow up study of 20 patients with schizophrenia that increased cortical thinning was found in middle frontal, middle and superior temporal gyrus in the context of stable neurocognitive performance and symptomatology. In the other longitudinal study of cortical thinning in schizophrenia, it was found that excessive cortical thinning occurred in bilateral temporal cortex and left frontal region [30].

Do putative psychosis susceptibility genes have a role in influencing these cortical thickness changes? A previous study which examined 48 patients with schizophrenia, 66 first-degree non-psychotic relatives of schizophrenia patients, 27 community probands and their 77 relatives replicated frontal and temporal thinning but suggested differential genetic influences in that genetic effects may be more prominent in medial temporal cortex compared with other cortical regions [31]. Dopamine related catechol-o-methyl transferase (COMT) val158met polymorphism has been implicated in cortical maturation processes [32] in that greater val allele dose was associated with greater cortical thinning in schizophrenia and their first degree relatives with persisting effects on dorsolateral prefrontal cortical thickness over time. Dystrobrevin binding protein 1 (DTNBP1) risk allele has also been associated with regional cortical thinning [33] in temporal brain regions within a study of 62 patients with schizophrenia and 42 healthy controls. In addition, functional D-amino oxidase activator (DAOA) gene risk variant Arg30Lys (rs2391191) has also been implicated in cortical thinning in schizophrenia [34]. 

 Whilst we found no difference in subcortical volumes amongst the four NRGN genotypes, thalamic shape abnormalities involving the posterior and medial-dorsal regions of the thalamus were found in schizophrenia with risk TT genotype. Specifically, our findings of absence of thalamic volume changes are consistent with some [35,36,37,38] but not all [39,40] earlier structural MRI studies examining thalamic volume changes in schizophrenia although not in context of genetic influences [41,42]. The thalamic shape abnormalities are consistent with studies which also found thalamic shape changes involving similar regions [43,44]. Csernansky et al. [43] used high dimensional brain mapping and reported thalamic shape changes in anterior and posterior extremes in their cohort of 52 patients with schizophrenia compared with 65 controls. We found in our earlier study that thalamic shape deformities included the anterior–medial and posterior–lateral aspects of the left thalamus, as well as the anterior–ventral aspect and medial body of the right thalamus in patients with early onset schizophrenia compared with controls [44], and which were correlated with poorer executive functioning and spatial working memory. Subsequent structural neuroimaging studies [45,46,47] also reported thalamic shape changes involving regions related to pulvinar nucleus and medial dorsal nucleus. 

The presence of cortical thinning alongside specific thalamic shape changes point towards the presence of thalamo-cortical morphological abnormalities in schizophrenia which may be partially mediated by NRGN. These data support extant theories of thalamo-cortical dysconnectivity in the pathophysiology of schizophrenia [48,49] and argue for specific involvement of differential thalamic networks implicating different thalamic nuclei in this illness. The reasons are at least threefold, First, this is consistent with previous work documenting topographical organization of connections as functionally parallel routes between particular cortical regions and thalamic nuclei [50], for example, prefrontal cortex is linked to the medial dorsal nucleus and the anterior nucleus of thalamus and visual association cortex is linked to the pulvinar nucleus. Second, there is neuropathological evidence of reductions of thalamic neurons involving specific medial dorsal nucleus [51,52] and pulvinar [53] as well as frontal cortical regions [54]. Third, these thalamo-cortical structural changes may underlie functional and cognitive changes as highlighted by recent studies which reported differential thalamo-cortical connectivity in resting state functional MRI investigations of schizophrenia [55] and decrease in total connectivity of thalamus to prefrontal cortex which correlated with working memory task [56].

The precise functions of NRGN and how it mediates cortical maturation and observed cortical thinning and thalamic shape abnormalities in schizophrenia are unclear at this juncture. It is known that NRGN regulates the local availability of Calmodulin (CaM) [2], which acts as a signaling hub to transmit Ca^2+^ ions in neurons [57]. Upon Ca^2+^ binding, Ca^2+^/CaM associates with a number of protein kinases that are critical for proper neural development [58] such as calcium/calmodulin-dependent protein kinase I, II and kinase (CaMKI, CaMKII and CaMKK). It has been shown that CaMKK activation of CaMKIα leads to axonal growth whereas CaMKK activation of CaMKIγ promotes dendritic outgrowth [59], hence CaMKI-CaMKK signaling may have direct impact on neuronal development, differentiation and synaptic plasticity [5]. There is evidence from one earlier study of reductions of NRGN proteins in prefrontal cortex of patients with schizophrenia [60]. Thus factors influencing the expression of NRGN have downstream effects on CaMKI, II and K signaling, and may subsequently impact on development of cortical matter and thalamus [7]. In addition, calmodulin activation of CaMKII strengthens NMDA receptor signalling [61]. Conversely, glutamate activation of NMDA receptors can lead to calclium influx into neurons and NRGN oxidation. NRGN, like DAOA, is involved in glutamate pathway regulation and may mediate effect of hypoglutamatergic function with impact on neural substrates in schizophrenia [62,63]. Furthermore, recent work has shown that neurotransmitter metabolism may be severely impaired in cortico-thalamic networks within MK-801 hypoglutamatergic animal model for schizophrenia, which is consistent with thalamo-cortical morphological abnormalities in schizophrenia [64]. Previous neuropathological studies in schizophrenia have considered cortical thinning to be the result of reduction or loss of cell number, cell density, or neuropil and observed that there are cell specific or layer specific changes in different cortical regions in schizophrenia [65,66]. Cortical thinning may also be related to altered minicolumn spacing and organisation within cortical regions that are related to neuroplastic changes or dendritic remodelling in the brain [67,68,69]. How these neuropathological changes are mediated by NRGN and inter-related putative genetic factors need further investigation. 

There are several limitations in this study. First, we did not perform analyses of other brain structural parameters such as specific white matter tracts that would provide further evidence of NRGN mediated connectivity disturbances in schizophrenia. Second, these findings need to be replicated in other larger samples. Third, we did not correlate the structural findings with neurocognitive data which would confer better insight into the full genetic impact of NRGN risk allele. However, the extant data of a study involving patients with schizophrenia suggested no association of NRGN with cognitive function in domains including general cognitive function, verbal working memory, spatial memory and attention [70]. Fourth, combinatorial approach with functional neuroimaging methods would provide further insights into the effect of NRGN on functional brain activations and how they relate to observed structural brain changes in cortical thickness and subcortical shape in schizophrenia. 

In conclusion, we found evidence for the influence of the genome wide supported psychosis vulnerability NRGN risk T genotype on thalamo-cortical morphology in schizophrenia involving widespread cortical thinning and thalamic shape abnormalities. These findings should stimulate further investigation into how these brain structural changes are related to alterations in brain function as well as the underlying NRGN mediated pathophysiological mechanisms. This may further unveil biological factors to allow better understanding of the genetic and neural basis of this potentially crippling neuropsychiatric condition.

## Supporting Information

File S1 **Figures S1 & S2.**
(DOC)

## References

[1] Lee KW, Woon PS, Teo YY, Sim K (2012) Genome wide association studies (GWAS) and copy number variation (CNV) studies of the major psychoses: what have we learnt? Neurosci Biobehav Rev 36: 556-571. doi:10.1016/j.neubiorev.2011.09.001. PubMed: 21946175. 21946175

[2] Stefansson H, Ophoff RA, Steinberg S, Andreassen OA, Cichon S et al. (2009) Common variants conferring risk of schizophrenia. Nature 460: 744-747. PubMed: 19571808. 19571808 10.1038/nature08186 PMC3077530

[3] de Arrieta CM, Jurado LP, Bernal J, Coloma A (1997) Structure, Organization, and Chromosomal Mapping of the Human Neurogranin Gene (< i> NRGN</i>). Genomics 41: 243-249 10.1006/geno.1997.4622 9143500

[4] Gaertner TR, Putkey JA, Waxham MN (2004) RC3/Neurogranin and Ca2+/calmodulin-dependent protein kinase II produce opposing effects on the affinity of calmodulin for calcium. J Biol Chem 279: 39374-39382. doi:10.1074/jbc.M405352200. PubMed: 15262982. 15262982

[5] Huang KP, Huang FL, Jäger T, Li J, Reymann KG et al. (2004) Neurogranin/RC3 enhances long-term potentiation and learning by promoting calcium-mediated signaling. J Neurosci 24: 10660-10669. doi:10.1523/JNEUROSCI.2213-04.2004. PubMed: 15564582. 15564582 PMC6730132

[6] Huang FL, Huang KP, Boucheron C (2007) Long-term enrichment enhances the cognitive behavior of the aging neurogranin null mice without affecting their hippocampal LTP. Learn Mem 14: 512-519. PubMed: 17671107. 17671107 10.1101/lm.636107 PMC1951789

[7] Pak JH, Huang FL, Li J, Balschun D, Reymann KG et al. (2000) Involvement of neurogranin in the modulation of calcium/calmodulin-dependent protein kinase II, synaptic plasticity, and spatial learning: a study with knockout mice. Proc Natl Acad Sci U S A 97: 11232-11237. doi:10.1073/pnas.210184697. PubMed: 11016969. 11016969 PMC17183

[8] Krug A, Krach S, Jansen A, Nieratschker V, Witt SH et al. (2013) The Effect of Neurogranin on Neural Correlates of Episodic Memory Encoding and Retrieval. Schizophr Bull, 39: 141–50. PubMed: 21799211. 21799211 10.1093/schbul/sbr076 PMC3523918

[9] Pohlack ST, Nees F, Ruttorf M, Witt SH, Nieratschker V et al. (2011) Risk variant for schizophrenia in the neurogranin gene impacts on hippocampus activation during contextual fear conditioning. Mol Psychiatry 16: 1072-1073. doi:10.1038/mp.2011.66. PubMed: 21647148. 21647148 PMC3199731

[10] Rose EJ, Morris DW, Fahey C, Robertson IH, Greene C et al. (2012) The Effect of the Neurogranin Schizophrenia Risk Variant rs12807809 on Brain Structure and Function. Twin Res Hum Genet 15: 296-303. doi:10.1017/thg.2012.7. PubMed: 22856365. 22856365

[11] Ohi K, Hashimoto R, Yasuda Y, Nemoto K, Ohnishi T et al. (2012) Impact of the Genome Wide Supported *NRGN* Gene on Anterior Cingulate Morphology in Schizophrenia. PLOS ONE 7: e29780. doi:10.1371/journal.pone.0029780. PubMed: 22253779. 22253779 PMC3257237

[12] Rose EJ, Donohoe G (2013) Brain vs Behavior: An Effect Size Comparison of Neuroimaging and Cognitive Studies of Genetic Risk for Schizophrenia. Schizophr Bull, 39: 518–26. PubMed: 22499782. 22499782 10.1093/schbul/sbs056 PMC3627766

[13] Winkler AM, Kochunov P, Blangero J, Almasy L, Zilles K et al. (2010) Cortical thickness or grey matter volume? The importance of selecting the phenotype for imaging genetics studies. NeuroImage 53: 1135-1146. doi:10.1016/j.neuroimage.2009.12.028. PubMed: 20006715. 20006715 PMC2891595

[14] Qiu A, Tuan TA, Woon PS, Abdul-Rahman MF, Graham S et al. (2010) Hippocampal-cortical structural connectivity disruptions in schizophrenia: An integrated perspective from hippocampal shape, cortical thickness, and integrity of white matter bundles. NeuroImage 52: 1181-1189. doi:10.1016/j.neuroimage.2010.05.046. PubMed: 20573561. 20573561

[15] Shenton ME, Dickey CC, Frumin M, McCarley RW (2001) A review of MRI findings in schizophrenia. Schizophr Res 49: 1–52. doi:10.1016/S0920-9964(01)00159-1. PubMed: 11343862. PMC2812015 11343862

[16] Sled JG, Zijdenbos AP, Evans AC (1998) A nonparametric method for automatic correction of intensity nonuniformity in MRI data. IEEE Trans Med Imaging 17: 87-97. doi:10.1109/42.668698. PubMed: 9617910. 9617910

[17] Qiu A, Brown T, Fischl B, Ma J, Miller MI (2010) Atlas generation for subcortical and ventricular structures with its applications in shape analysis. IEEE Trans Image Process 19: 1539-1547. doi:10.1109/TIP.2010.2042099. PubMed: 20129863. 20129863 PMC2909363

[18] Fischl B, Dale AM (2000) Measuring the thickness of the human cerebral cortex from magnetic resonance images. Proc Natl Acad Sci U S A 97: 11050-11055. doi:10.1073/pnas.200033797. PubMed: 10984517. 10984517 PMC27146

[19] Zhong J, Qiu A (2010) Multi-manifold diffeomorphic metric mapping for aligning cortical hemispheric surfaces. NeuroImage 49: 355-365. doi:10.1016/j.neuroimage.2009.08.026. PubMed: 19698793. 19698793

[20] Chung MK, Robbins SM, Dalton KM, Davidson RJ, Alexander AL et al. (2005) Cortical thickness analysis in autism with heat kernel smoothing. NeuroImage 25: 1256-1265. doi:10.1016/j.neuroimage.2004.12.052. PubMed: 15850743. 15850743

[21] Chung MK, Worsley KJ, Nacewicz BM, Dalton KM, Davidson RJ (2010) General multivariate linear modeling of surface shapes using SurfStat. NeuroImage 53: 491-505. doi:10.1016/j.neuroimage.2010.06.032. PubMed: 20620211. 20620211 PMC3056984

[22] Crespo-Facorro B, Roiz-Santiáñez R, Pérez-Iglesias R, Rodriguez-Sanchez JM, Mata I et al. (2011) Global and regional cortical thinning in first-episode psychosis patients: relationships with clinical and cognitive features. Psychol Med 41: 1449-1460. doi:10.1017/S003329171000200X. PubMed: 20942995. 20942995 PMC3954972

[23] Takayanagi Y, Takahashi T, Orikabe L, Mozue Y, Kawasaki Y et al. (2011) Classification of First-Episode Schizophrenia Patients and Healthy Subjects by Automated MRI Measures of Regional Brain Volume and Cortical Thickness. PLOS ONE 6: e21047. doi:10.1371/journal.pone.0021047. PubMed: 21712987. 21712987 PMC3119676

[24] Kuperberg GR, Broome MR, McGuire PK, David AS, Eddy M et al. (2003) Regionally localized thinning of the cerebral cortex in schizophrenia. Arch Gen Psychiatry 60: 878–888. doi:10.1001/archpsyc.60.9.878. PubMed: 12963669. 12963669

[25] Gutiérrez-Galve L, Wheeler-Kingshott CAM, Altmann DR, Price G, Chu EM et al. (2010) Changes in the frontotemporal cortex and cognitive correlates in first-episode psychosis. Biol Psychiatry 68: 51-60. doi:10.1016/j.biopsych.2010.03.019. PubMed: 20452574. 20452574 PMC3025327

[26] Schultz CC, Koch K, Wagner G, Roebel M, Schachtzabel C et al. (2010) Reduced cortical thickness in first episode schizophrenia. Schizophr Res 116: 204–209. doi:10.1016/j.schres.2009.11.001. PubMed: 19926451. 19926451

[27] Oertel-Knöchel V, Knöchel C, Rotarska-Jagiela A, Reinke B, Prvulovic D et al. (2013) Association between psychotic symptoms and cortical thickness reduction across the schizophrenia spectrum. Cerebral Cortex, 23: 61–70. PubMed: 22291030. 22291030 10.1093/cercor/bhr380

[28] Rimol LM, Hartberg CB, Nesvåg R, Fennema-Notestine C, Hagler DJ Jr. et al. (2010) Cortical thickness and subcortical volumes in schizophrenia and bipolar disorder. Biol Psychiatry 68: 41-50. doi:10.1016/j.biopsych.2010.03.036. PubMed: 20609836. 20609836

[29] Cobia DJ, Smith MJ, Wang L, Csernansky JG (2012) Longitudinal progression of frontal and temporal lobe changes in schizophrenia. Schizophr Res 139: 1-6. doi:10.1016/j.schres.2012.05.002. PubMed: 22647883. 22647883 PMC3413315

[30] van Haren NEM, Schnack HG, Cahn W, van den Heuvel MP, Lepage C et al. (2011) Changes in cortical thickness during the course of illness in schizophrenia. Arch Gen Psychiatry 68: 871–880. doi:10.1001/archgenpsychiatry.2011.88. PubMed: 21893656. 21893656

[31] Yang Y, Nuechterlein KH, Phillips O, Hamilton LS, Subotnik KL et al. (2010) The contributions of disease and genetic factors towards regional cortical thinning in schizophrenia: the UCLA family study. Schizophr Res 123: 116-125. doi:10.1016/j.schres.2010.08.005. PubMed: 20817413. 20817413 PMC2988766

[32] Raznahan A, Greenstein D, Lee Y, Long R, Clasen L et al. (2011) Catechol-o-methyl transferase (COMT) val< sup> 158</sup> met polymorphism and adolescent cortical development in patients with childhood-onset schizophrenia, their non-psychotic siblings, and healthy controls. NeuroImage 57: 1517-1523. doi:10.1016/j.neuroimage.2011.05.032. PubMed: 21620981. 21620981 PMC3285479

[33] Narr KL, Szeszko PR, Lencz T, Woods RP, Hamilton LS et al. (2009) DTNBP1 is associated with imaging phenotypes in schizophrenia. Hum Brain Mapp 30: 3783-3794. doi:10.1002/hbm.20806. PubMed: 19449336. 19449336 PMC3176807

[34] Schultz CC, Nenadic I, Koch K, Wagner G, Roebel M et al. (2011) Reduced cortical thickness is associated with the glutamatergic regulatory gene risk variant DAOA Arg30Lys in schizophrenia. Neuropsychopharmacology 36: 1747-1753. doi:10.1038/npp.2011.56. PubMed: 21508934. 21508934 PMC3138664

[35] Ananth H, Popescu I, Critchley HD, Good CD, Frackowiak RS et al. (2002) Cortical and subcortical gray matter abnormalities in schizophrenia determined through structural magnetic resonance imaging with optimized volumetric voxel-based morphometry. Am J Psychiatry 159: 1497-1505. doi:10.1176/appi.ajp.159.9.1497. PubMed: 12202269. 12202269

[36] Kemether EM, Buchsbaum MS, Byne W, Hazlett EA, Haznedar M et al. (2003) Magnetic resonance imaging of mediodorsal, pulvinar, and centromedian nuclei of the thalamus in patients with schizophrenia. Arch Gen Psychiatry 60: 983-991. doi:10.1001/archpsyc.60.9.983. PubMed: 14557143. 14557143

[37] Salgado-Pineda P, Baeza I, Pérez-Gómez M, Vendrell P, Junqué C et al. (2003) Sustained attention impairment correlates to gray matter decreases in first episode neuroleptic-naive schizophrenic patients. NeuroImage 19: 365-375. doi:10.1016/S1053-8119(03)00094-6. PubMed: 12814586. 12814586

[38] Gilbert AR, Rosenberg DR, Harenski K, Spencer S, Sweeney JA et al. (2001) Thalamic volumes in patients with first-episode schizophrenia. Am J Psychiatry 158: 618-624. doi:10.1176/appi.ajp.158.4.618. PubMed: 11282698. 11282698

[39] Bagary MS, Foong J, Maier M, duBoulay G, Barker GJ et al. (2002) A magnetization transfer analysis of the thalamus in schizophrenia. J Neuropsychiatry Clin Neurosci 14: 443-448. doi:10.1176/appi.neuropsych.14.4.443. PubMed: 12426413. 12426413

[40] Bridle N, Pantelis C, Wood SJ, Coppola R, Velakoulis D et al. (2002) Thalamic and caudate volumes in monozygotic twins discordantfor schizophrenia. Aust N Z J Psychiatry 36: 347-354. doi:10.1046/j.1440-1614.2001.01022.x. PubMed: 12060183. 12060183

[41] Cronenwett WJ, Csernansky J (2010) Thalamic pathology in schizophrenia. Curr. Top - Behav Neuroscience 4: 509-528. doi:10.1007/7854_2010_55. 21312411

[42] Sim K, Cullen T, Ongur D, Heckers S (2006) Testing models of thalamic dysfunction in schizophrenia using neuroimaging. J Neural Transm 113: 907-928. doi:10.1007/s00702-005-0363-8. PubMed: 16252070. 16252070

[43] Csernansky JG, Schindler MK, Splinter NR, Wang L, Gado M et al. (2004) Abnormalities of thalamic volume and shape in schizophrenia. Am J Psychiatry 161: 896-902. doi:10.1176/appi.ajp.161.5.896. PubMed: 15121656. 15121656

[44] Qiu A, Zhong J, Graham S, Chia MY, Sim K (2009) Combined analyses of thalamic volume, shape and white matter integrity in first-episode schizophrenia. NeuroImage 47: 1163-1171. doi:10.1016/j.neuroimage.2009.04.027. PubMed: 19375511. 19375511

[45] Coscia DM, Narr KL, Robinson DG, Hamilton LS, Sevy S et al. (2009) Volumetric and shape analysis of the thalamus in first-episode schizophrenia. Hum Brain Mapp 30: 1236-1245. doi:10.1002/hbm.20595. PubMed: 18570200. 18570200 PMC6870587

[46] Kang DH, Kim SH, Kim CW, Choi JS, Jang JH et al. (2008) Thalamus surface shape deformity in obsessive-compulsive disorder and schizophrenia. Neuroreport 19: 609-613. doi:10.1097/WNR.0b013e3282fa6db9. PubMed: 18382272. 18382272

[47] Smith MJ, Wang L, Cronenwett W, Mamah D, Barch DM et al. (2011) Thalamic morphology in schizophrenia and schizoaffective disorder. J Psychiatr Res 45: 378-385. doi:10.1016/j.jpsychires.2010.08.003. PubMed: 20797731. 20797731 PMC2996474

[48] Jones EG (1997) Cortical development and thalamic pathology in schizophrenia. Schizophr Bull 23: 483-501. doi:10.1093/schbul/23.3.483. PubMed: 9327511. 9327511

[49] Swerdlow NR (2010) Behavioral neurobiology of schizophrenia and its treatment. Preface. Curr. Top - Behav Neuroscience 4: v-vii. 21312394

[50] Alexander GE, DeLong MR, Strick PL (1986) Parallel organization of functionally segregated circuits linking basal ganglia and cortex. Annu Rev Neurosci 9: 357-381. doi:10.1146/annurev.ne.09.030186.002041. PubMed: 3085570. 3085570

[51] Pakkenberg B (1990) Pronounced reduction of total neuron number in mediodorsal thalamic nucleus and nucleus accumbens in schizophrenics. Arch Gen Psychiatry 47: 1023-1028. doi:10.1001/archpsyc.1990.01810230039007. PubMed: 2241504. 2241504

[52] Young KA, Manaye KF, Liang C, Hicks PB, German DC (2000) Reduced number of mediodorsal and anterior thalamic neurons in schizophrenia. Biol Psychiatry 47: 944-953. doi:10.1016/S0006-3223(00)00826-X. PubMed: 10838062. 10838062

[53] Byne W, Buchsbaum MS, Mattiace LA, Hazlett EA, Kemether E et al. (2002) Postmortem assessment of thalamic nuclear volumes in subjects with schizophrenia. Am J Psychiatry 159: 59-65. doi:10.1176/appi.ajp.159.1.59. PubMed: 11772691. 11772691

[54] Volk DW, Lewis DA (2010) Prefrontal cortical circuits in schizophrenia. Curr. Top - Behav Neuroscience 4: 485-508. doi:10.1007/7854_2010_44. 21312410

[55] Woodward ND, Karbasforoushan H, Heckers S (2012) Thalamocortical dysconnectivity in schizophrenia. Am J Psychiatry 169: 1092-1099. doi:10.1176/appi.ajp.2012.12010056. PubMed: 23032387. 23032387 PMC3810300

[56] Marenco S, Stein JL, Savostyanova AA, Sambataro F, Tan HY et al. (2012) Investigation of anatomical thalamo-cortical connectivity and FMRI activation in schizophrenia. Neuropsychopharmacology 37: 499-507. doi:10.1038/npp.2011.215. PubMed: 21956440. 21956440 PMC3242311

[57] Díez-Guerra FJ (2010) Neurogranin, a link between calcium/calmodulin and protein kinase C signaling in synaptic plasticity. IUBMB Life 62: 597-606. doi:10.1002/iub.357. PubMed: 20665622. 20665622

[58] Zheng JQ, Poo MM (2007) Calcium signaling in neuronal motility. Annu Rev Cell Dev Biol 23: 375-404. doi:10.1146/annurev.cellbio.23.090506.123221. PubMed: 17944572. 17944572

[59] Ageta-Ishihara N, Takemoto-Kimura S, Nonaka M, Adachi-Morishima A, Suzuki K et al. (2009) Control of cortical axon elongation by a GABA-driven Ca2+/calmodulin-dependent protein kinase cascade. J Neurosci 29: 13720-13729. doi:10.1523/JNEUROSCI.3018-09.2009. PubMed: 19864584. 19864584 PMC2796271

[60] Broadbelt K, Ramprasaud A, Jones LB (2006) Evidence of altered neurogranin immunoreactivity in areas 9 and 32 of schizophrenic prefrontal cortex. Schizophr Res 87: 6-14. doi:10.1016/j.schres.2006.04.028. PubMed: 16797925. 16797925

[61] Hayashi Y (2009) Long-term potentiation: two pathways meet at neurogranin. EMBO J 28: 2859-2860. doi:10.1038/emboj.2009.273. PubMed: 19809472. 19809472 PMC2760120

[62] Li J, Pak JH, Huang FL, Huang KP (1999) N-methyl-D-aspartate induces neurogranin/RC3 oxidation in rat brain slices. J Biol Chem 274: 1294-1300. doi:10.1074/jbc.274.3.1294. PubMed: 9880498. 9880498

[63] Tsai G, Coyle JT (2002) Glutamatergic mechanisms in schizophrenia. Annu Rev Pharmacol Toxicol 42: 165-179. doi:10.1146/annurev.pharmtox.42.082701.160735. PubMed: 11807169. 11807169

[64] Eyjolfsson EM, Nilsen LH, Kondziella D, Brenner E, Håberg A et al. (2011) Altered 13C glucose metabolism in the cortico-striato-thalamo-cortical loop in the MK-801 rat model of schizophrenia. J Cereb Blood Flow Metab 31: 976-985. doi:10.1038/jcbfm.2010.193. PubMed: 21081956. 21081956 PMC3063632

[65] Harrison PJ (1999) The neuropathology of schizophrenia. A critical review of the data and their interpretation. Brain 122 ( 4): 593-624. doi:10.1093/brain/122.4.593. 10219775

[66] Heckers S (1997) Neuropathology of schizophrenia. Schizophr Bull 23: 403-421. doi:10.1093/schbul/23.3.403. PubMed: 9327506. 9327506

[67] Arendt T (2004) Neurodegeneration and plasticity. Int J Dev Neurosci 22: 507-514. doi:10.1016/j.ijdevneu.2004.07.007. PubMed: 15465280. 15465280

[68] Chance SA, Casanova MF, Switala AE, Crow TJ, Esiri MM (2006) Minicolumn thinning in temporal lobe association cortex but not primary auditory cortex in normal human ageing. Acta Neuropathol 111: 459-464. doi:10.1007/s00401-005-0014-z. PubMed: 16496164. 16496164

[69] Rakic P (1995) A small step for the cell, a giant leap for mankind: a hypothesis of neocortical expansion during evolution. Trends Neurosci 18: 383-388. doi:10.1016/0166-2236(95)93934-P. PubMed: 7482803. 7482803

[70] Donohoe G, Walters J, Morris DW, Da Costa A, Rose E et al. (2011) A neuropsychological investigation of the genome wide associated schizophrenia risk variant NRGN rs12807809. Schizophr Res 125: 304-306. doi:10.1016/j.schres.2010.10.019. PubMed: 21112188. 21112188

