# Supplementary material for: Effects of the Neurogranin Variant rs12807809 on Thalamocortical Morphology in Schizophrenia
Source: PLoS One. 2013 Dec 30;8(12):e85603. doi: 10.1371/journal.pone.0085603 (PMC3875583; doi:10.1371/journal.pone.0085603)
Supplement: File S1 — Figures S1 & S2. (DOC) [file pone.0085603.s001.doc]

**
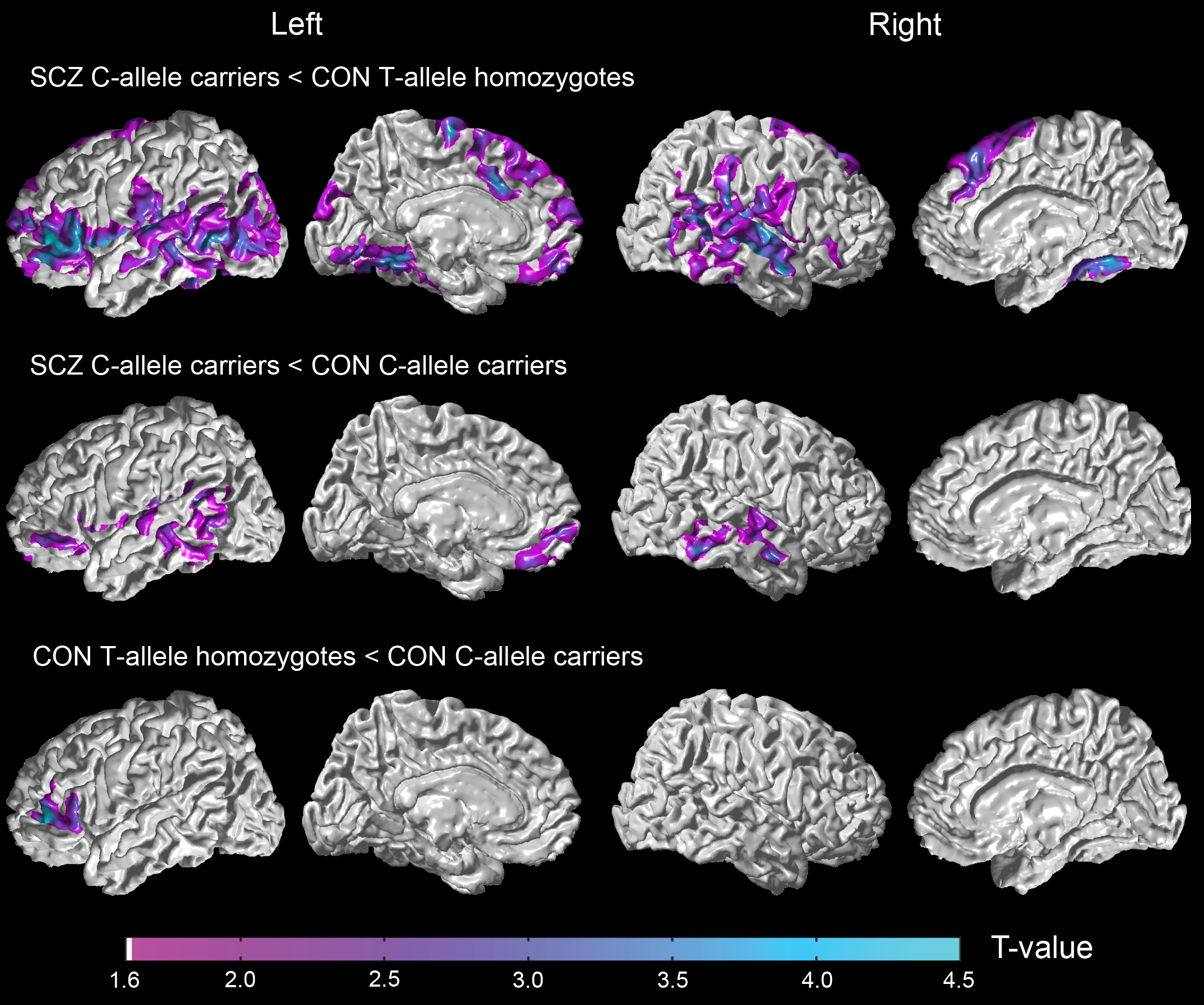
**

**Figure S1.** Statistical maps of cortical thickness differences between schizophrenia C-allele carriers and: control T-allele homozygotes (top panel); schizophrenia C-allele carriers and control C-allele carriers (middle panel); control T-allele homozygotes and control C-allele carriers (bottom panel). T-values are shown only in the regions with significant group differences after correction for multiple comparisons. Keys: SCZ – Schizophrenia; CON – Control.

**
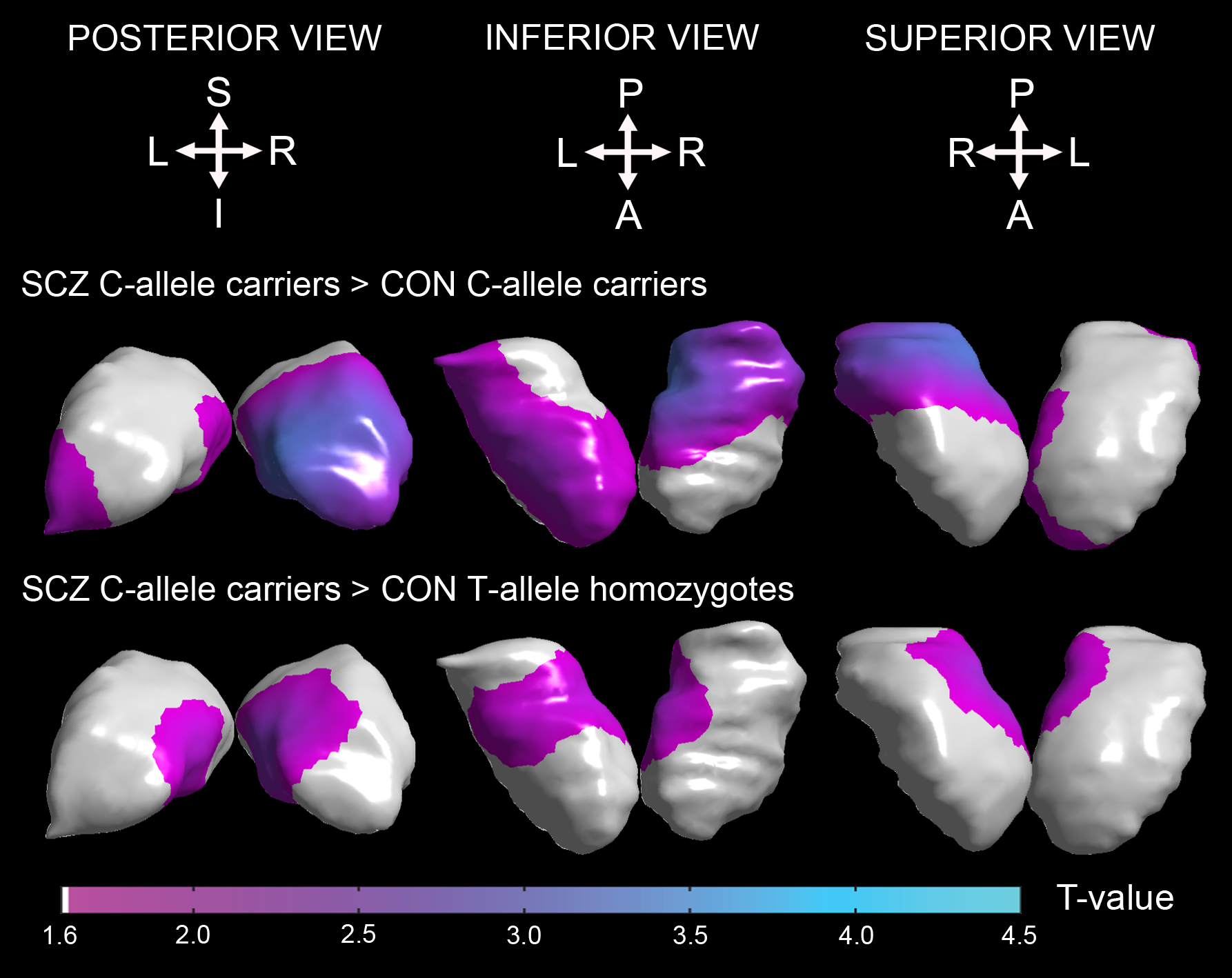
**

**Figure S2.** Statistical maps of thalamic shape differences between schizophrenia C-allele carriers and control C-allele carriers (top panel); schizophrenia C-allele carriers and control T-allele homozygotes (bottom panel). T-values are shown only in the regions with significant group differences after the correction of multiple comparisons. Keys: S – superior; I – inferior; A – anterior; P – posterior; L – left; R – right.
